# Klotho overexpression improves amyloid‐β clearance and cognition in the APP/PS1 mouse model of Alzheimer's disease

**DOI:** 10.1111/acel.13239

**Published:** 2020-09-21

**Authors:** Yue Zhao, Chen‐Ye Zeng, Xiao‐Hong Li, Ting‐Ting Yang, Xi Kuang, Jun‐Rong Du

**Affiliations:** ^1^ Department of Pharmacology, Key Laboratory of Drug‐Targeting and Drug Delivery System of the Education Ministry and Sichuan Province, Sichuan Engineering Laboratory for Plant‐Sourced Drug and Sichuan Research Center for Drug Precision Industrial Technology, West China School of Pharmacy Sichuan University Chengdu China

**Keywords:** Alzheimer's disease, Aβ clearance, Klotho

## Abstract

Alzheimer's disease (AD) is the most prevalent type of dementia, characterized by the presence of amyloid‐β (Aβ) plaques. We previously reported that Klotho lowered Aβ levels in the brain and protected against cognitive deficits in amyloid precursor protein/presenilin 1(APP/PS1) mice. However, the underlying mechanism remains unclear. In this study, we induced intracerebral Klotho overexpression in 13‐month‐old APP/PS1 mice by injecting lentivirus that carried full‐length mouse Klotho cDNA in the lateral ventricle of the brain. We examined the effects of Klotho overexpression on cognition, Aβ burden, Aβ‐related neuropathology, microglia transformation, and Aβ transport systems in vivo. Additionally, we investigated the effects of Klotho on Aβ transport at the blood–cerebrospinal fluid barrier by knocking down Klotho in primary human choroid plexus epithelial cells (HCPEpiCs). The upregulation of Klotho levels in the brain and serum significantly ameliorated Aβ burden, neuronal and synaptic loss and cognitive deficits in aged APP/PS1 mice. Klotho treatment significantly inhibited NACHT, LRR, and PYD domain‐containing protein 3 (NLRP3) and the subsequent transformation of microglia to the M2 type that may enhance microglia‐mediated Aβ clearance. Meanwhile, Klotho overexpression also regulated Aβ transporter expression, which may promote Aβ transporter‐mediated Aβ clearance. Moreover, the ability of HCPEpiCs to transport Aβ in vitro was also significantly impaired by Klotho knockdown. Given the neuroprotective effect of Klotho overexpression, the present findings suggest that Klotho should be further investigated as a potential therapeutic target for AD.

## Graphical abstract

*Klotho*, as an anti‐aging gene, has been studied in the field of AD in recent years. Our data showed that Klotho overexpression inhibited NLRP3 inflammasome activation and promoted Aβ clearance through an increase in M2 type microglia and the regulation of Aβ transporters in APP/PS1 mice, which effectively relieved neuroinflammation and Aβ burden and ameliorated AD‐like phenotypes.

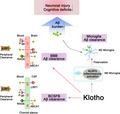

## 1 INTRODUCTION

Alzheimer's disease (AD) is a chronic age‐related neurodegenerative disease that is mainly characterized by senile plaque deposition, intracellular neurofibrillary tangles, and progressive cognitive decline (Masters et al., 2015). As of 2018, more than 50 million cases of AD were diagnosed worldwide. Various hypotheses have been proposed to explain the etiology and pathogenesis of Alzheimer's disease. The amyloid‐β (Aβ) cascade hypothesis is the mainstream hypothesis that has been used over the past two decades to explain the pathogenesis of AD (Hardy & Higgins, 1992). An imbalance of the production and clearance of Aβ peptide is observed in the brains of AD patients, which subsequently form amyloid plaques and cause neuronal dysfunction (Selkoe & Hardy, 2016).

The deficiency of Aβ clearance rather than Aβ overproduction should be considered a major causal factor, which in turn may trigger neuroinflammation, progressive synaptic loss, and ultimately cognitive decline (Nalivaeva & Turner, 2019). Studies have shown that the activation of NACHT, LRR, and PYD domain‐containing protein 3 (NLRP3) inflammasome by Aβ mediates the transformation of microglia from the M2 type to M1 type, reducing the clearance of Aβ (Frautschy et al., 1998; Heneka et al., 2013). Thus, a vicious feedback loop is formed between Aβ and activation of the NLRP3 inflammasome, leading to progressive deterioration (Sheedy et al., 2013). Alterations of the expression or activity of efflux transporters (e.g., low‐density lipoprotein receptor‐related protein 1 [LRP1], P‐glycoprotein [P‐gp], and adenosine triphosphate [ATP]‐binding cassette transporter A1 [ABCA1]) and the influx transporter receptor for advanced glycation end products (RAGE) were observed in AD patients and mouse models, resulting in impairments in the clearance of Aβ (Chiu et al., 2015; Elali & Rivest, 2013). Inhibiting the NLRP3/caspase‐1 axis and enhancing transporter‐mediated Aβ clearance may theoretically prevent the development of AD.

The anti‐aging gene *Klotho* is highly expressed in the kidneys and choroid plexus, with lower expression in brain parenchyma (Kuro‐o et al., 1997; Kurosu et al., 2005; Lim et al., 2015). Klotho has various physiological functions, such as anti‐neuroinflammatory effects and the regulation of autophagy (Bian, Neyra, Zhan, & Hu, 2015; Xu & Sun, 2015). Previous studies showed that Klotho in the brain of AD patients and mouse models was significantly lower than in age‐matched healthy groups (Dubal et al., 2015; Kuang et al., 2017; Massó et al., 2015; Semba et al., 2014). More researches have provided indications that Klotho may alleviate an array of neurodegenerative diseases (Jamali‐Raeufy et al., 2017; Dubal et al., 2015; Scazzone et al., 2019; Zeldich et al., 2019). These findings provide convincing evidence that Klotho may be a novel therapeutic target for the treatment of AD. In a previous study, we maintained intracerebral Klotho upregulation in 7‐month‐old amyloid precursor protein/presenilin 1 (APP/PS1) mice for up to 3 months, which significantly reduced Aβ levels and attenuated cognitive dysfunction （Zeng et al., 2019). However, the effects of long‐term Klotho upregulation on the late stage of AD and the underlying mechanism remain unclear.

Based on accumulating evidence of anti‐AD effects of Klotho, we hypothesized that Klotho ameliorates Aβ pathology and cognitive impairment by promoting the transformation of microglia from M1 type to M2 type and regulating Aβ transporter function. In the present study, we maintained Klotho overexpression for 6 months in APP/PS1 mice and evaluated the role of the NLRP3 inflammasome in microglia transformation and Aβ transporter expression at the blood–brain barrier (BBB) and blood–cerebrospinal fluid (CSF) barrier. We also knocked down Klotho in human primary choroid plexus epithelial cells (HCPEpiCs) and evaluated the effect of Klotho on Aβ transport clearance across the blood–CSF barrier.

## 2 RESULTS

### 2.1 Klotho overexpression attenuated cognitive impairment in aged APP/PS1 mice

**FIGURE 1:**
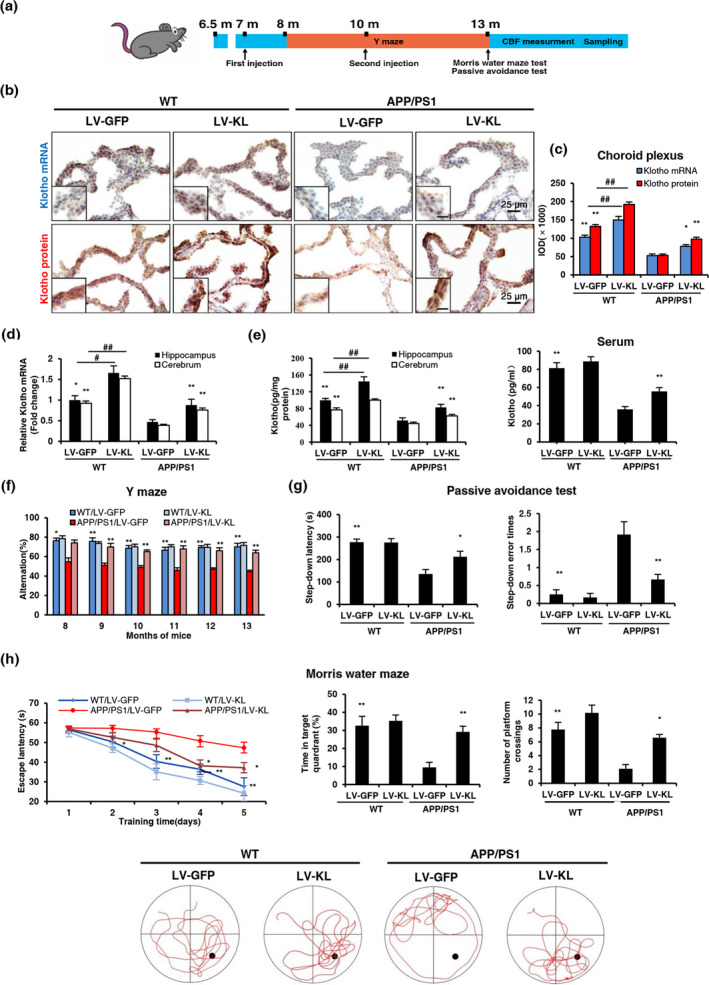
Klotho overexpression attenuated cognitive impairment in aged APP/PS1 mice. (a) Temporal schematic diagram of the experimental procedures. (b) Representative images of Klotho mRNA and protein levels in the choroid plexus, detected by in situ hybridization and immunohistochemistry, respectively. The inset shows representative overall staining intensity. Scale bar = 10 μm. (c) Quantitative image analysis of Klotho mRNA and protein levels based on the integrated optical density (IOD) of positive immunostaining (brown). (d) Analysis of Klotho mRNA levels in the hippocampus and cerebrum by quantitative real‐time polymerase chain reaction (qRT‐PCR). Relative Klotho mRNA levels were normalized to GAPDH and are expressed as fold changes relative to the WT/LV‐GFP group. (e) Klotho levels in the hippocampus, cerebrum and serum were measured by enzyme‐linked immunosorbent assay (ELISA). (f) Percentage of alternation in the Y‐maze test. (g) Latency and number of errors in the passive avoidance test. (h) Escape latency, time in the target quadrant, number of platform crossings, and characteristic swimming trails in the Morris water maze. *n* = 12/group, except *n* = 6/group in (d, e). The data are expressed as mean ± standard error of mean (*SEM*). The statistical analysis was performed using two‐way analysis of variance (ANOVA) and mixed‐design ANOVA (f, escape latencies in h) followed by the Bonferroni‐Holm post hoc test. *p < 0.05, **p < 0.01, vs. APP/PS1/LV‐GFP group; ^##^ *p* < 0.01, vs. WT/LV‐GFP group

We first measured Klotho levels in the brain and serum when mice were 13 months old. Compared with the WT/LV‐GFP group (WT, wide type; LV, lentivirus; GFP, green fluorescent protein), we observed a ~50% decrease in Klotho mRNA and protein levels in the choroid plexus and cerebrum in the APP/PS1/LV‐GFP group. Klotho expression was significantly upregulated by LV‐KL (KL, *Klotho*) in the cerebrum in WT mice and APP/PS1 mice (Figure 1b‐e). Since cognitive function is closely related to hippocampus, we simultaneously measured the Klotho levels in hippocampus and found that similar to the trend of cerebrum, LV‐KL also increased the mRNA and protein levels of Klotho in the hippocampus in WT mice and APP/PS1 mice (Figure 1b‐e). Notably, LV‐KL reversed the reduction of serum Klotho levels in APP/PS1 mice (Figure 1e). One possible way by which Klotho enters the blood is through the integrity loss of the BBB in APP/PS1 mice (Ahn et al., 2018; Takechi et al., 2010; Zhang et al., 2018). The current findings showed that LV‐KL significantly upregulated Klotho expression in the brain in both WT and AD mice and upregulated serum Klotho level in AD mice.

Three neurobehavioral tests were performed to evaluate cognitive function. No significant differences were found between the WT/LV‐GFP and WT/LV‐KL groups. In the Y‐maze (Figure 1f), the percentage of alternation in the APP/PS1/LV‐GFP group was significantly lower than in the WT/LV‐GFP group beginning at 8 months of age, which was significantly reversed by LV‐KL. In the passive avoidance test (Figure 1g), the step‐down latency significantly decreased, and the step‐down error times significantly increased in the APP/PS1/LV‐GFP group compared with the WT/LV‐GFP group, and these changes were significantly reversed by LV‐KL. In the Morris water maze (Figure 1h), the escape latency was significantly longer in the APP/PS1/LV‐GFP group than in the WT/LV‐GFP group, whereas the percent time in the target quadrant and number of platform crossings significantly decreased in the APP/PS1/LV‐GFP group compared with the WT/LV‐GFP group. Notably, LV‐KL significantly improved these behavioral impairments in APP/PS1 mice. The current findings showed that Klotho overexpression ameliorated cognitive deficits in AD mice.

### 2.2 Klotho overexpression decreased Aβ burden in aged APP/PS1 mice

**FIGURE 2:**
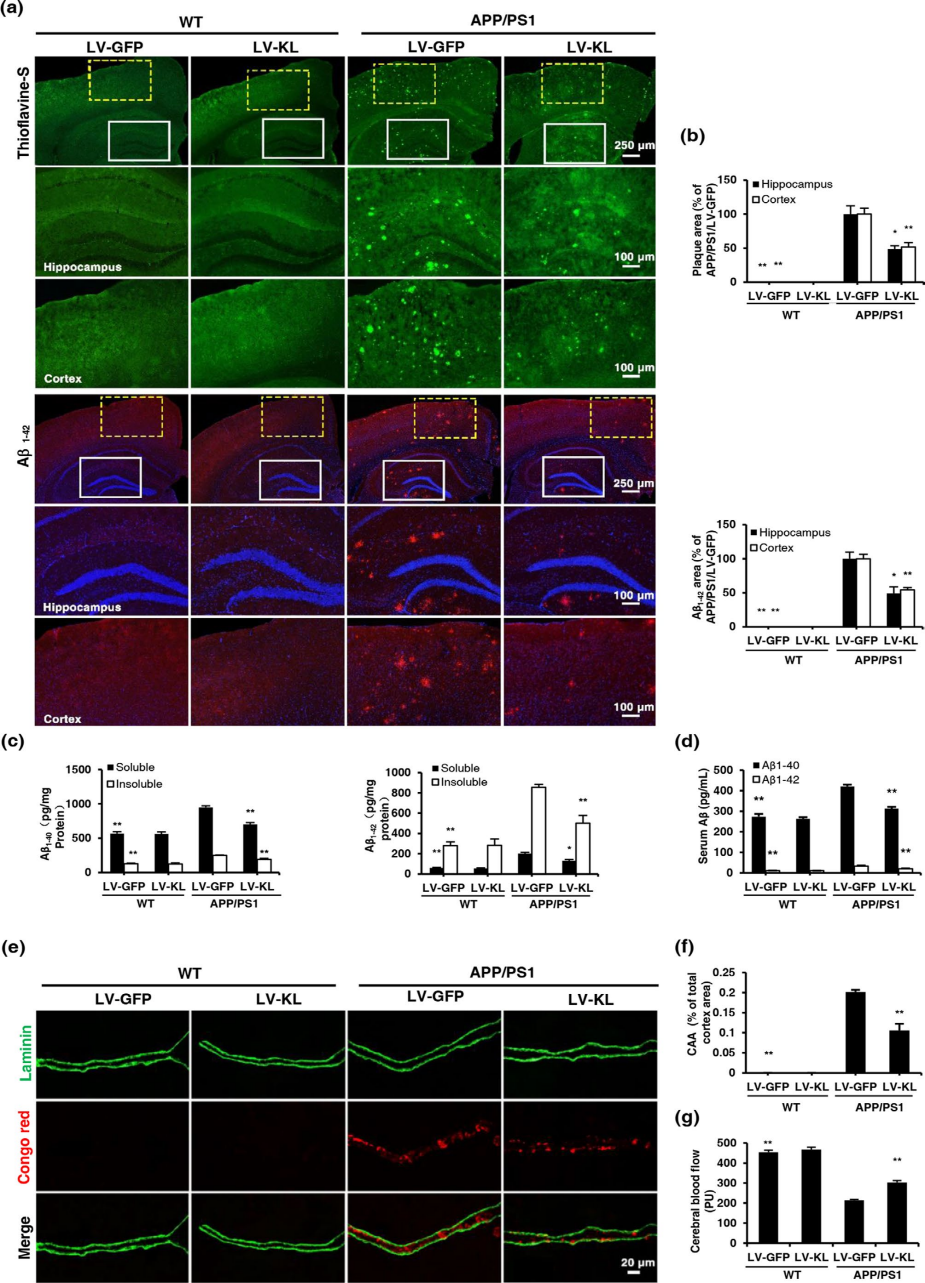
Klotho overexpression reduced Aβ burden in aged APP/PS1 mice. (a) The distribution of amyloid plaques was detected using Thioflavine‐S staining and Aβ_1‐42_ monoclonal antibody in the hippocampus and cortex. (b) Quantitative image analysis of amyloid plaque accumulation based on Thioflavine‐S‐positive and Aβ_1‐42_‐positive fluorescence area in the hippocampus and cortex. The data are expressed as each normalized value relative to the APP/PS1/LV‐GFP group. (c) Levels of soluble and insoluble Aβ_1‐40_ and Aβ_1‐42_ in brain homogenates, measured by ELISA. (d) Serum levels of Aβ_1‐40_ and Aβ_1‐42_, measured by ELISA. (e) Representative images of cerebral amyloid angiopathy (CAA), revealed by double‐stained vessels (Laminin, green; aggregated Aβ peptide, Congo red). (f) Quantitative image analysis of CAA load in the total cerebral cortex area. (g) Cerebral blood flow (CBF) in the cortex. *n* = 6/group, except *n* = 12 in (g). The data are expressed as mean ± *SEM*. The statistical analysis was performed using two‐way ANOVA followed by the Bonferroni‐Holm post hoc test. *p < 0.05, **p < 0.01, vs. APP/PS1/LV‐GFP group

The accumulation of Aβ results in AD lesions in APP/PS1 mice. We examined brain, serum, and cerebrovascular levels of Aβ in mice. No amyloid plaques were observed in WT mice. LV‐KL treatment resulted in a 40%–50% reduction of Aβ plaques in the hippocampus and cortex in APP/PS1 mice (Figure 2a,b). LV‐KL significantly decreased the levels of soluble and insoluble Aβ_1‐40_ and Aβ_1‐42_ in the brain in the APP/PS1/LV‐GFP group (Figure 2c). Serum levels of soluble Aβ_1‐40_ and Aβ_1‐42_ in the APP/PS1/LV‐GFP group were also reduced by LV‐KL (Figure 2d).

Failure of the brain to clear excessive Aβ may lead to vascular Aβ deposition and subsequently cause cerebral amyloid angiopathy (CAA) (Greenberg et al., 2020). Double‐staining for Aβ (red) and laminin (green) elucidated the effects of Klotho on CAA. No apparent vascular amyloid deposition was detected in WT mice. However, cortical CAA was observed in APP/PS1/LV‐GFP mice, which was significantly downregulated by LV‐KL (Figure 2e,f).

The current findings showed that Klotho overexpression decreased Aβ burden in AD mice. Notably, consistent with the previously reported interactions between cerebral blood flow (CBF) reductions and aberrant vascular Aβ deposition, we observed a significant reduction of CBF in APP/PS1/LV‐GFP mice compared with WT/LV‐GFP mice, and this reduction was significantly reversed by LV‐KL (Figure 2g).

### 2.3 Klotho overexpression ameliorated neuronal injury in the brain in aged APP/PS1 mice

**FIGURE 3:**
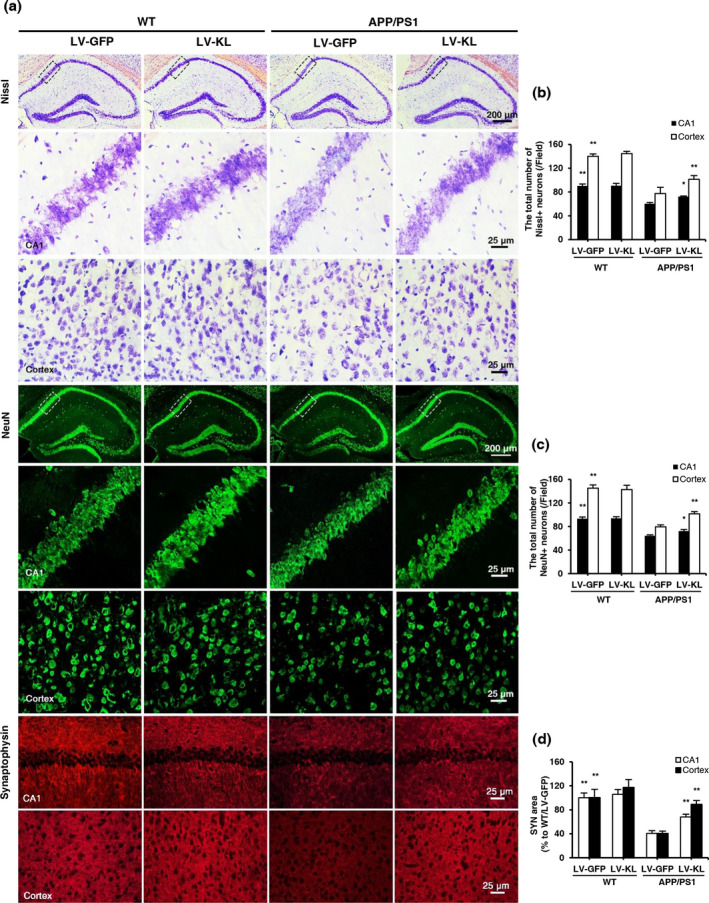
Klotho overexpression alleviated neuronal injury in the brain in aged APP/PS1 mice. (a) Representative images of Nissl staining, NeuN, and synaptophysin immunofluorescent staining in the hippocampal CA1 area and cortex. (b) Quantitative analysis of the number of Nissl‐positive neurons in the hippocampal CA1 area and cortex. (c) Quantitative analysis of the number of NeuN‐positive neurons in the hippocampal CA1 area and cortex. (d) Quantitative analysis of the synaptophysin (SYN)‐positive area in the hippocampal CA1 area and cortex. *n* = 6/group. The data are expressed as mean ± *SEM*. The statistical analysis was performed using two‐way ANOVA followed by the Bonferroni‐Holm post hoc test. **p < 0.01, vs. APP/PS1/LV‐GFP group

Amyloid‐β is highly neurotoxic, which may result in cerebral neuronal injury in vivo. In the present study, we observed approximately 30% and 50% neuronal loss in the hippocampal CA1 area and cortex, respectively, in APP/PS1/LV‐GFP mice compared with WT/LV‐GFP mice, while LV‐KL significantly preserved neuronal survival in APP/PS1 mice (Figure 3a‐c). In addition, significantly reduced synaptophysin (SYN) level was observed in the hippocampus CA1 area and cortex in APP/PS1/LV‐GFP mice compared with WT/LV‐GFP mice, which was significantly rescued by LV‐KL (Figure 3a,d). The current findings showed that Klotho overexpression ameliorated neuronal and synaptic loss in AD mice.

### 2.4 Klotho overexpression promoted microglial transformation and inhibited tau pathology in the brain in aged APP/PS1 mice

**FIGURE 4:**
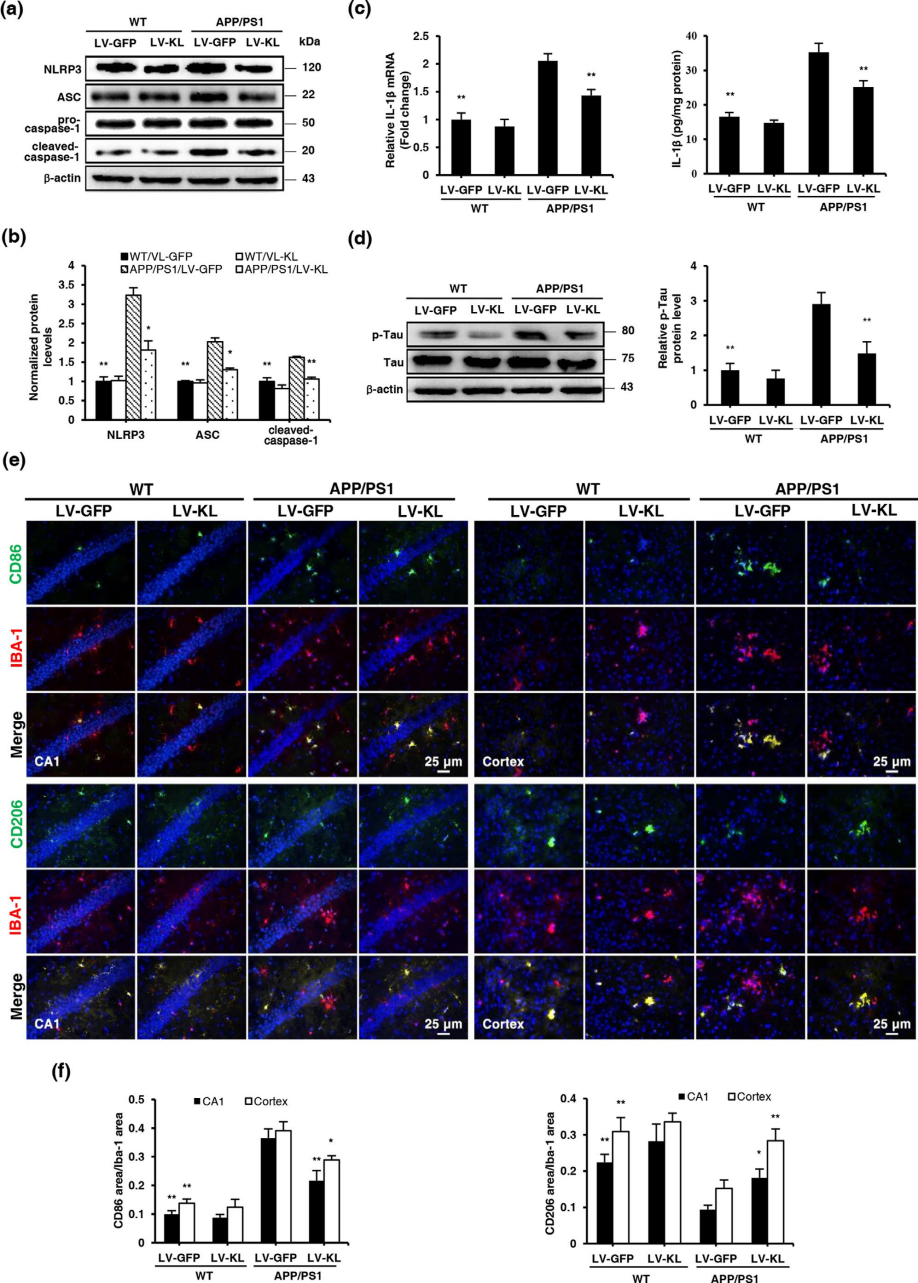
Klotho overexpression promoted microglial transformation and alleviated Tau pathology in the brain in aged APP/PS1 mice. (a, b) Representative Western blotting and quantification of NLRP3, ASC, cleaved caspase‐1, and β‐actin in brain tissues. The amount of NLRP3, ASC, and cleaved caspase‐1 were normalized to β‐actin. (c) Analyses of IL‐1β mRNA levels and protein concentrations in brain homogenates by qRT‐PCR and ELISA, respectively. Relative mRNA levels of IL‐1β were normalized to GAPDH and are expressed as fold changes relative to the WT/LV‐GFP group. (d) Representative Western blotting and quantification of p‐Tau and Tau in brain tissues. The amount of p‐Tau was normalized to Tau. (e) Representative images of CD86 and CD206 (green) counterstained with Iba‐1 (red) and nuclear DNA staining of DAPI (blue) in the hippocampal CA1 area and cortex. (f) Quantitative image analysis of CD86/Iba‐1 and CD206/Iba‐1 expression based on positive fluorescence area in the hippocampal CA1 area and cerebral cortex. The data are expressed as each normalized value relative to the WT/LV‐GFP group. *n* = 6/group, except *n* = 4/group in (a, b, d). The data are expressed as mean ± *SEM*. The statistical analysis was performed using two‐way ANOVA followed by the Bonferroni‐Holm post hoc test. *p < 0.05, **p < 0.01, vs. APP/PS1/LV‐GFP group

Studies have shown that Aβ activates the NLRP3 inflammasome. We examined activation of the NLRP3/caspase‐1 signaling pathway in the brain in APP/PS1 mice. Western blotting showed that the expression of NLRP3, apoptosis‐associated speck‐like protein (ASC), and cleaved caspase‐1 significantly increased in the brain in APP/PS1/LV‐GFP mice compared with WT/LV‐GFP controls (Figure 4a,b), and these increases were significantly reversed by LV‐KL. The mRNA and protein levels of interleukin‐1β (IL‐1β), the final product of the NLRP3/caspase‐1 signaling pathway, also significantly increased in APP/PS1 mice compared with WT mice, and this increase was reversed by LV‐KL (Figure 4c). The current findings showed that Klotho overexpression inhibited activation of the NLRP3/caspase‐1 signaling pathway. We also observed that LV‐KL decreased tau phosphorylation, indicating that LV‐KL improved tau pathology (Figure 4d).

Microglia are highly plastic and can switch between the classic activated M1 type and substitutively activated M2 type. The NLRP3/caspase‐1 signaling pathway is one of the main pathways that regulate microglial activation and transformation. Studies have shown that the NLRP3 inflammasome promotes the transformation of microglia from the M2 type to M1 type, triggering an inflammatory response and reducing the clearance of Aβ. Therefore, we detected the surface antigens CD86 and CD206 of these two phenotypes in microglia. APP/PS1/LV‐GFP mice exhibited more than a two‐fold increase in the CD86‐to‐Iba‐1 ratio and an approximately 50% decrease in the CD206‐to‐Iba‐1 ratio in the hippocampal CA1 area and cortex compared with WT/LV‐GFP controls. Interestingly, LV‐KL induced an approximately 30% decrease in the CD86‐to‐Iba‐1 ratio and 90% increase in the CD206‐to‐Iba‐1 ratio in the hippocampal CA1 area and cortex in APP/PS1 mice compared with WT/LV‐GFP mice (Figure 4e,f).

The current findings showed that Klotho overexpression promoted the transformation of microglia from the M1 type to M2 type in AD mice, which was associated with inhibition of the NLRP3/caspase‐1 signaling pathway.

### 2.5 Klotho overexpression affected the expression of Aβ transporters in aged APP/PS1 mice

**FIGURE 5:**
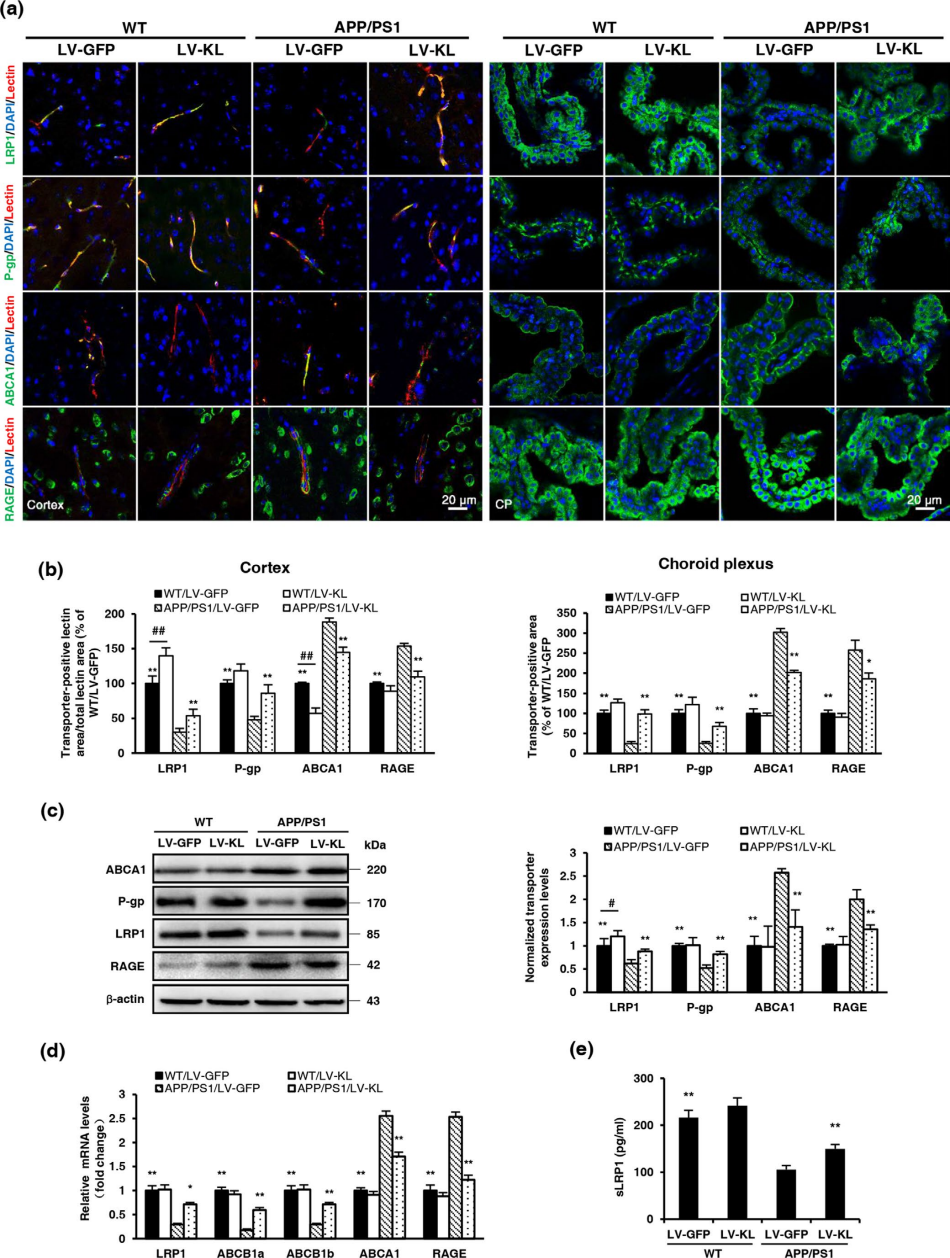
Klotho overexpression affected the expression levels of Aβ transporters in aged APP/PS1 mice. (a) Representative immunofluorescence images of Aβ transporters (green) in the cortex (left) and choroid plexus (right), including low‐density lipoprotein receptor‐related protein 1 (LRP1), P‐glycoprotein (P‐gp), ATP‐binding cassette transporter A1 (ABCA1), and receptor for advanced glycation end products (RAGE). The lectin‐positive area (red) indicates micro‐vessels. Nuclei were stained with DAPI (blue). (b) Quantitative analysis of the levels of Aβ transporters in micro‐vessels and the choroid plexus. (c) Representative Western blotting and quantification of the relative protein levels of LRP1, P‐gp, ABCA1, and RAGE in brain tissue homogenates. Protein levels were normalized to GAPDH and are expressed as fold changes relative to the WT/LV‐GFP group. (d) Analyses of LRP1, P‐gp, ABCA1, and RAGE mRNA levels in brain tissue homogenates by qRT‐PCR. Relative mRNA levels were normalized to GAPDH and are expressed as fold changes relative to the WT/LV‐GFP group. (e) Soluble LRP1 (sLRP1) levels in serum, measured by ELISA. *n* = 6/group, except *n* = 4/group in (c). The data are expressed as mean ± *SEM*. The statistical analysis was performed using two‐way ANOVA followed by the Bonferroni‐Holm post hoc test. *p < 0.05, **p < 0.01, vs. APP/PS1/LV‐GFP group; ^#^ *p* < 0.05, ^##^ *p* < 0.01, vs. WT/LV‐GFP group

The continuous clearance of Aβ from the central nervous system is vital for preventing its accumulation. Transport from the brain to the blood through the BBB and blood–CSF barrier primarily accounts for the clearance of total Aβ. LRP1 and P‐gp might act in a coordinated fashion to traffic Aβ peptides from the brain into systemic circulation. Additionally, RAGE was shown to transport Aβ peptides back into the ISF (Xin, Tan, Cao, Yu, & Tan, 2018). In the present study, LRP1 and P‐gp protein levels significantly decreased and ABCA1 and RAGE protein levels significantly increased in lectin‐positive endothelial cells in the cortex and choroid plexus in APP/PS1/LV‐GFP mice compared with WT/LV‐GFP mice, which were reversed by Klotho treatment (Figure 5a,b). The mRNA and protein levels of LRP1 and P‐gp were upregulated, and ABCA1 and RAGE levels were downregulated by LV‐KL in brain homogenates in APP/PS1 mice (Figure 5c,d).

The current findings showed that Klotho overexpression regulated the expression of Aβ transporters. Notably, Klotho overexpression enhanced the level of soluble LRP1 (sLRP1) in serum of APP/PS1 mice (Figure 5e).

### 2.6 Klotho knockdown decreased Aβ transport across the human blood–CSF barrier in an in vitro model and regulated the expression of Aβ transporters in HCPEpiCs

**FIGURE 6:**
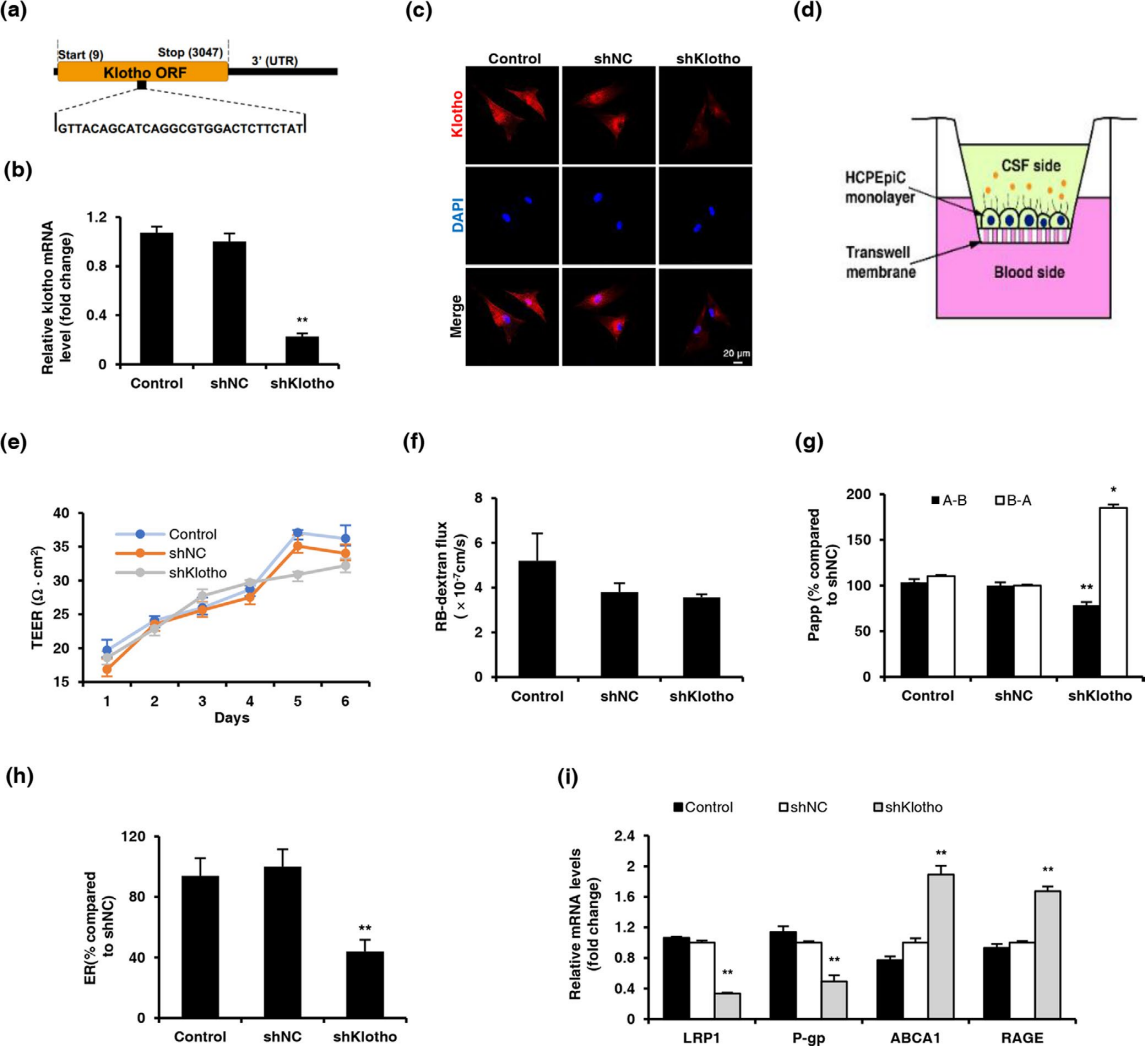
Klotho knockdown decreased the active transport of Aβ across the human blood–CSF barrier in an in vitro model. (a) Schematic representation of the location of the shRNA target within the Klotho coding sequence (shKlotho). (b) Analysis of Klotho mRNA levels in primary human choroid plexus epithelial cells (HCPEpiCs) infected with shKlotho or shNC at a multiplicity of infection (MOI) of 80. Relative mRNA levels were normalized to β‐actin and are expressed as fold changes relative to shNC‐treated cells. (c) Representative confocal images of Klotho (red) in HCPEpiCs, examined by immunofluorescence 5 days after infection. Nuclear DNA was stained with DAPI (blue). (d) Paradigm for the in vitro model of the human blood–cerebrospinal fluid barrier (BCSFB), consisting of a monolayer of HCPEpiCs. (e) Transepithelial electrical resistance (TEER) measured after seeding across the BCSFB monolayer. (f) Permeability of HCPEpiCs monolayer to 1 mg/ml RB‐dextran. (g) Bidirectional transfer of 0.5 μM FITC‐Aβ_1‐42_ across the BCSFB monolayer from the basolateral to apical side (B → A) and from the apical to basolateral side (A → B). (h) FITC‐Aβ_1‐42_ efflux rate across the BCSFB monolayer. The data were normalized to shNC‐treated cells. (i) Analyses of LRP1, P‐gp, ABCA1, and RAGE mRNA levels in primary human choroid plexus epithelial cells by qRT‐PCR. The data were normalized to β‐actin and are expressed as fold changes relative to shNC‐treated cells. The data are from three independent experiments and are expressed as mean ± *SEM*. *p < 0.05, **p < 0.01, vs. shNC‐treated cells. Cells that were incubated in epithelial cell medium without vectors were used as the normal control. The statistical analysis was performed using one‐way ANOVA followed by the LSD test. Kruskal–Wallis test was used when variance was uneven

To further investigate the involvement of Klotho in Aβ trafficking at the blood–CSF barrier, we silenced Klotho in HCPEpiCs by infecting these cells with a lentivirus that expressed shRNA that targeted Klotho mRNA at 1414–1442 bp (shKlotho; Figure 6a), which significantly reduced Klotho expression by approximately 77.4% (Figure 6b,c). We then constructed an in vitro model of the blood–CSF barrier by culturing a single monolayer of HCPEpiCs on inserts with collagen‐coated transwell membrane filters (Figure 6d). Consistent with previous studies (Kuplennik, Lang, Steinfeld, & Sosnik, 2019), our blood–CSF barrier model had a high TEER value (>30 Ω · cm^2^) and a low permeability constant (5.19 ± 1.23 ⋅ 10^−7^ cm/s) to rhodamine B (RB)‐labeled dextran, indicating a relatively impermeable monolayer (Figure 6e,f). The Aβ_1‐42_ transport analysis showed that shKlotho significantly decreased Papp_A‐B_, increased Papp_B‐A_, and decreased the rate of efflux of soluble FITC‐Aβ_1‐42_ (Figure 6g,h), suggesting that Klotho knockdown impeded Aβ clearance across the human blood–CSF barrier.

Next, we investigated whether the reduction of Aβ efflux across the blood–CSF barrier by Klotho knockdown was associated with alterations of Aβ transporter levels. We examined mRNA levels of transporters in each group. We observed significant decreases in LRP1 and P‐gp mRNA levels and significant increases in ABCA1 and RAGE mRNA levels in the shKlotho group compared with the shNC group (Figure 6i). The current findings showed that Klotho knockdown inhibited Aβ transport across the blood–CSF barrier by regulating the expression of its transporters in choroid plexus epithelial cells.

## 3 DISCUSSION

*Klotho* was initially described as an anti‐aging gene. Previous studies showed that Klotho expression in the brain in AD patients and mouse models significantly decreased compared with age‐matched control groups. Klotho overexpression has been shown to alleviate AD‐like pathology and cognitive impairments in mouse models of AD (Dubal et al., 2015; Kuang et al., 2017; Massó et al., 2015; Semba et al., 2014). The present study used 7‐month‐old APP/PS1 mice, which developed early AD‐relevant pathological deficits, and maintained Klotho overexpression for up to 6 months by lentivirus injections. Our data showed that Klotho overexpression attenuated AD pathology, which was consistent with our previous report (Zeng et al., 2019). We found that Klotho overexpression significantly improved cognitive decline, reduced Aβ burden, ameliorated neuronal and synaptic loss, and increased CBF in APP/PS1 mice. These neuroprotective effects of Klotho appeared to be relevant to the promotion of the transformation of microglia from the M1 type to M2 type and the regulation of Aβ transporters, which increased the clearance of Aβ. Notably, we did not observe similar effects in WT mice, suggesting that Klotho overexpression may have little effect in normal mice. However, we also observed that Klotho overexpression had no effect on the content of p53 in the brain of APP/PS1 mice (Figure S1), suggesting that Klotho upregulation does not affect the expression of other proteins, such as p53.

It is well known that Aβ‐induced neurotoxic effects (neuronal and synaptic loss) are the potential causes of cognitive deficits in AD. A recent study published in *Molecular Psychiatry* indicates that the treatment with adipoRon (APN receptor agonist) might significantly lower Aβ levels, rescue neuronal and synaptic loss, and improve spatial memory functions in AD mice (Ng et al., 2020). SYN is an important indicator of synaptic density and synaptic formation. In the present study, i.c.v. injection of LV‐KL increased NeuN and SYN immunoreactivity, and improved cognitive function in AD mice. Our results showed that treatment with Klotho might ameliorate Aβ‐induced neuronal and synaptic loss, and cognitive deficits in mice. In addition, several studies have reported the effects of Klotho on synaptic plasticity. Dubal et al. (2014) found that elevating Klotho in mice might increase synaptic levels of the GluN2B subunit of NMDARs, enhance hippocampal LTP, and increase the GluN2B portion of NMDAR currents. Dubal et al. (2015) also showed that Klotho elevation in hAPP/KL mice increased NMDAR‐dependent long‐term potentiation (LTP) despite of not altering hippocampal levels of Aβ. Li et al. (2019) found that secreted human Klotho altered some synaptic plasticity‐related proteins and mediated a significant increase in fEPSP slope per unit of stimulation intensity in mice. These results showed the direct regulation of Klotho itself on synaptic plasticity in mice. The further investigation is required to elucidate the underlying mechanism of Klotho in the future.

Aβ is an NLRP3 inflammasome activator. Numerous studies have shown that the NLRP3 inflammasome plays an important role in the pathogenesis of AD (Saresella et al., 2016; Yin et al., 2018). Consistent with previous studies (Zhu, Stein, et al., 2018), we observed significant increases in the expression of NLRP3, ASC, and cleaved caspase‐1 in the brain in APP/PS1 mice compared with WT mice, which were reversed by LV‐KL. Klotho overexpression also significantly inhibited the expression of IL‐1β, a product of the activation of NLRP3/caspase‐1 signaling pathway. These results indicated the inhibition of the NLRP3/caspase‐1 pathway by Klotho overexpression. Interestingly, a recent study reported that NLRP3 activation by Aβ rather than Aβ itself was closely related to tau phosphorylation (Ising et al., 2019). In the present study, we also found a decrease in tau phosphorylation levels in the brain in APP/PS1 mice with Klotho overexpression.

Promoting the clearance of Aβ is an important topic in AD research. Aβ clearance in the central nervous system is a complex process that is mediated by various systems and cell types, including the ubiquitin‐proteasome system, autophagy‐lysosome, proteases, microglial phagocytosis, glymphatic drainage and transport to the periphery via the BBB, arachnoid villi, and the blood–CSF barrier (Xin et al., 2018). Our previous study focused on the impact of Klotho overexpression on the degradation of autophagy for Aβ clearance (Zeng et al., 2019). However, whether Klotho overexpression also affects other pathways remains unclear. Previous studies showed that the inhibition of NLRP3 inflammasome promoted the transformation of microglia to M2 type and enhanced the clearance of Aβ as well as spatial memory in AD mice (Heneka et al., 2013). Since Klotho can reduce NLRP3/caspase‐1 signaling pathway, we speculated that Klotho can promote the transformation of microglia to M2 type. Consistent with the conjecture, we observed that the overexpression of Klotho increased the expression of CD206 in the hippocampal CA1 area and cortex of APP/PS1 mice, which is the surface antigen of M2‐type microglia. In general, the present findings showed that Klotho overexpression promoted the transformation of microglia in APP/PS1 mice from the M1 type to M2 type, thereby increasing the clearance of Aβ by microglia in AD mice.

In addition to microglial phagocytosis, Aβ trafficking from the brain to blood by transporters at the BBB plays an important role in reducing Aβ levels in the brain (Xin et al., 2018). Cross‐talk among transporters, including LRP1 and P‐gp, plays a critical role in promoting Aβ clearance from the brain to blood (ElAli & Rivest, 2013), and RAGE mediates the influx of Aβ back into the brain (Cai et al., 2016). Consistent with previous articles (Chiu et al., 2015), we found that LRP1 and P‐gp expression decreased, and RAGE expression increased in the BBB and cerebrum in APP/PS1 mice. Our results showed that Klotho overexpression restored the aberrant levels of these transporters. Klotho overexpression induced the expression of LRP1 and P‐gp, suggesting upregulation of the transcytosis of Aβ peptide across the BBB from the brain to blood. The reduction of RAGE expression suggested suppression of the influx of Aβ peptide across the BBB from the blood to brain. Collectively, our findings indicated that Klotho overexpression upregulated Aβ clearance across the BBB. Notably, we also found that Klotho overexpression decreased the expression of ABCA1 in the BBB and brain in AD mice. ABCA1 has been reported to facilitate Aβ elimination in an ApoE‐dependent manner, and ApoE lipidation may competes for LRP1, blocking Aβ clearance (ElAli & Rivest, 2013; Martiskainen et al., 2013). In the present study, we did not investigate ApoE. Whether the LRP1‐mediated transport of ApoE‐bound Aβ is affected by Klotho overexpression is unknown.

The sLRP1 is a cleavage product of LRP1 that sequesters Aβ in the periphery and mediates its clearance in the liver. It has been reported that sLRP1 can prevent the access of Aβ to RAGE and RAGE‐mediated Aβ influx (Wang, Gu, Masters, & Wang, 2017). To further investigate whether alterations of serum Aβ levels were related to disturbances in periphery transport, we measured circulating sLRP1 levels. We found that serum sLRP1 levels significantly decreased in APP/PS1 mice, which was reversed by Klotho. The upregulation of sLRP1 by Klotho would further enhance peripheral Aβ sequestration, thereby promoting Aβ clearance in the periphery.

Aβ in the brain is mainly removed by efflux across the BBB, and Aβ in the extracellular space of the brain can enter CSF freely. Aβ in CSF can be absorbed into the circulation via spinal arachnoid villi or the blood–CSF barrier, and the blood–CSF barrier is the primary channel for Aβ removal (Xin et al., 2018). Capillaries in the choroid plexus are highly fenestrated. The blood–CSF barrier is formed by choroid plexus epithelial cells (CPECs) and separates blood from CSF. Additionally, CPECs contain many specific transporters that confer an active role in the regulation of transport between the blood and CSF (Erickson & Banks, 2013). In the present study, we observed the distribution of these transporters by immunofluorescence microscopy. We found that LRP1 and RAGE moved between the cytosol and external limiting membranes of the choroid plexus. ABCA1 was clearly localized to the outer edge of the choroid plexus, whereas P‐gp was expressed at the basolateral membrane of CPECs. These findings suggested that transport/distribution principles of transporters at the BBB were also applicable to the blood–CSF barrier in removing Aβ from CSF. We observed decreases in expression of the efflux transporters LRP1 and P‐gp and increases in the expression of ABCA1 and RAGE at the blood–CSF barrier in 13‐month‐old APP/PS1 mice, suggesting that compensatory Aβ clearance via the blood–CSF barrier failed similarly to failure at the BBB. Together, our findings suggested that Klotho overexpression promoted Aβ clearance across the blood–CSF barrier by increasing LRP1 and P‐gp expression and decreasing RAGE and ABCA1 expression in the choroid plexus in APP/PS1 mice.

The results of our in vitro model of the human blood–CSF barrier also directly showed that Klotho facilitated Aβ efflux across the blood–CSF barrier by regulating transporter levels. We found that Klotho knockdown in the in vitro blood–CSF barrier model significantly decreased the transepithelial permeability of soluble Aβ_1‐42_ from the CSF side to blood side but increased its permeability from the blood side to CSF side, suggesting that Klotho promoted Aβ clearance from the blood–CSF barrier. Klotho knockdown also decreased LRP1 and P‐gp mRNA levels but increased ABCA1 and RAGE mRNA levels in HCPEpiCs, consistent with the in vivo results. Overall, our results provided evidence that Klotho plays a role in Aβ clearance by regulating Aβ transporters at the blood–CSF barrier. The production of CSF and integrity of the blood–CSF barrier were also shown to play important roles in Aβ clearance. Dysfunction of the choroid plexus, together with compromised CSF production (Chiu et al., 2012), diminishes the clearance of Aβ peptide, indicating its involvement in AD pathology (Serot et al., 2011; Silverberg et al., 2010). In the present study, we did not observe significant effects of Klotho knockdown on blood–CSF barrier integrity, reflected by no changes in TEER or dextran permeability.

In summary, the present study found that Klotho overexpression inhibited NLRP3 inflammasome activation and promoted Aβ clearance through an increase in M2 type microglia and the regulation of Aβ transporters in APP/PS1 mice, which effectively relieved neuroinflammation and Aβ burden and ameliorated AD‐like phenotypes. Moreover, Klotho knockdown reduced the transporter‐mediated efflux rate of soluble Aβ_1‐42_ across the blood–CSF barrier in an in vitro monolayer model. Our findings indicate that Klotho overexpression in the central nervous system may be a potential strategy for the treatment of AD.

## 4 EXPERIMENTAL PROCEDURES

### 4.1 Cell cultures

HCPEpiCs were obtained from ScienCell Research Laboratories and cultured in epithelial cell medium according to the distributor's recommendations. HCPEpiCs at passages 3–5 were used for the experiments. HEK293 cells were purchased from Humanitas Clinical and Research Center (Rozzano, Milan, Italy) and cultured in Dulbecco's modified Eagle's medium supplemented with 10% fetal bovine serum.

### 4.2 Animals

Male 6.5‐month‐old APP^swe^/PS1^dE9^ transgenic mice (Beijing HFK Bioscience Co. Ltd) and WT mice both on C57BL/6 background were used in this study. All treatments were approved by the ethics committee of the University of Sichuan (number K2018085). All studies were carried out in accordance with the Regulations of Experimental Animal Administration promulgated by the State Committee of Science and Technology of China.

### 4.3 Plasmid and lentivirus preparation

Green fluorescent protein (GFP)‐tagged lentiviral plasmid carrying a full‐length mouse *Klotho* cDNA (LV‐KL) or shRNA against human Klotho (shKlotho) were purchased from OriGene Technologies, supplemented with a negative control (LV‐GFP or shNC, respectively). The mouse lentiviral particles were subsequently packaged as we previously reported (Zeng et al., 2019). The shRNA lentiviral particles were packaged by GeneChem Company.

### 4.4 Lentivirus administration

Seven‐month‐old mice were randomly divided into four groups: APP/PS1/LV‐GFP, APP/PS1/LV‐KL, WT/LV‐GFP, and WT/LV‐KL (*n* = 12 per group). Then, a bilateral intracerebroventricular (i.c.v.) injection of corresponding LV‐KL or LV‐GFP (3 µl per side, 4.10 × 10^7^ TU/ml) was conducted as reported previously. Three months later, one additional injection of lentivirus was given in the same way.

### 4.5 Neurobehavioral tests

The Y‐maze test was carried out on the last day of each month after i.c.v. injection. The passive avoidance test and Morris water maze test were conducted 3 months after supplemental injection. All the neurobehavioral tests were performed as we previously reported (Zeng et al., 2019).

### 4.6 Cerebral blood flow measurement

One day after Morris water maze test, the mice were anesthetized and fixed, and cerebral blood flow (CBF) in the cortex was measured by moorVMS laser Doppler perfusion (Moor Instruments Ltd) as we previously described (Zeng et al., 2019).

### 4.7 Tissue and blood collection

After CBF measurement, blood was collected from orbital sinus immediately for serum separation. Followed by transcardially perfused with cold saline, the brains were rapidly removed and split into two hemispheres. The right hemisphere was frozen for 20‐micron‐thick sections, while the left hemisphere was homogenized for biochemical analysis without choroid plexus.

### 4.8 Histological evaluation

The in situ hybridization (ISH) analysis, immunohistochemistry (IHC) analysis and immunofluorescence (IF) analysis were performed as we previously reported (Zeng et al., 2019). Negative control sections were processed by omitting the complementary RNA or primary antibody. Nissl staining (0.5% cresyl violet) and Congo red staining (0.5% Congo red) were performed the same as previous studies (Zeng et al., 2019). For Thioflavine‐S staining, the slides were incubated with 0.05% Thioflavine‐S (Sigma) and then differentiated in 80% ethanol solution. Each section containing one microscopic field of the choroid plexus was digitized using a 40× objective. The total area of CAA was analyzed as percentage of total cortex area, and seven different sections from each mouse were determined in CAA. For other histological evaluation, three microscopic fields in hippocampus or cortex of each section were examined, respectively. All slides were mounted and imaged on a Nikon microscope or ZEISS LSM800. IF analysis of cultured cells was performed in the same way as tissue sections. The primary antibodies we used were showed in Table 1.

**TABLE 1:** Primary antibodies used in this study

Antibody	Application	Dilution rate	Source	Catalog number
Klotho	IHC	1:200	Abcam, UK	ab181373
Klotho	ICC/IF	1:100	Santa Cruz, USA	sc‐515939
NeuN	IF	1:100	Abcam, UK	ab177487
SYN	IF	1:100	Santa Cruz, USA	sc‐17750
Aβ_1-42_	IF	1:200	Abcam, UK	ab201060
Laminin	IF	1:100	Abcam, UK	ab11757
Iba‐1	IF	1:500	Wako, Japan	019‐19741
CD86	IF	1:50	Abcam, UK	ab119857
CD206	IF	1:50	Abcam, UK	ab8918
NLRP3	WB	1:1000	AdipoGen, USA	AG‐20B‐0014‐C100
Caspase‐1	WB	1:1000	AdipoGen, USA	AG‐20B‐0044‐C100
ASC	WB	1:1000	Wanlei, China	WL02462
LRP1	WB, IF	1:100	Abcam, UK	ab28320
RAGE	WB, IF	1:200	Santa Cruz, USA	sc‐365154
ABCA1	WB, IF	1:200	Novus, USA	NB400‐105S
P‐gp	WB, IF	1:100	Santa Cruz, USA	sc‐55510
Lectin	IF	1:100	Vector Laboratories, USA	DL‐1177
Tau	WB	1:1000	Abcam, UK	Ab80579
p‐Tau	WB	1:1000	CST, USA	#9632
β‐actin	WB	1:1000	Zhongshanjinqiao, China	TA‐09

Abbreviations: IF, Immunofluorescence; IHC, Immunohistochemistry; WB, Western blotting.

### 4.9 Quantitative real‐time polymerase chain reactions

Total RNA was isolated using TRIzol reagent (Invitrogen) and cDNA was generated using RevertAid First Strand cDNA Synthesis kit (Thermo). The optimized primer sequences were synthesized by Beijing Genomics Institute (Table 2). Quantitative real‐time polymerase chain reactions (qRT‐PCR) was carried out as we previously reported (Zeng et al., 2019). The results were normalized to GAPDH using the 2^−ΔΔCT^ method.

**TABLE 2:** Nucleotide sequences of primers used for the quantitative real‐time PCR experiments

Gene	Primer sequence forward (5′−3′)	Primer sequence reverse (5′−3′)
*Mouse GAPDH*	AGCGAGACCCCACTAACATC	GGTTCACACCCATCACAAAC
*Mouse IL‐1β*	TTCAAATCTCGCAGCAGCAC	GTGCAGTTGTCTAATGGGAACG
*Mouse Klotho*	GGCTTTCCTCCTTTACCTGAAAA	CACATCCCACAGATAGACATTCG
*Mouse LRP1*	CCGACTGGCGAACAAATACAC	ATCGGCTTTGTTGCACGTG
*Mouse RAGE*	AAAACGACAACCCAGGCGT	ATTCTCTGGCATCTCCGCTTC
*Mouse ABCA1*	GCCTGGATCTACTCTGTCGC	GCCATTGTCCAGACCCATGA
*Mouse ABCB1a*	CATTGCGATAGCAGGAGTGG	CACCAAGTAGGCACCGAACC
*Mouse ABCB1b*	CTGGTATGGGACATCCTTGGT	CTGTCTGGTTTGTAGCCCTTTG
*Human β‐actin*	ACGAGGCCCAGAGCAAGAG	GGTGTGGTGCCAGATCTTCTC
*Human klotho*	GCTCTCAAAGCCCACATACTG	GCAGCATAACGATAGAGGCC
*Human LRP1*	ACGAGGCCCAGAGCAAGAG	GGTGTGGTGCCAGATCTTCTC
*Human RAGE*	GTGTCCTTCCCAACGGCTC	ATTGCCTGGCACCGGAAAA
*Human ABCA1*	GAACTGGCTGTGTTCCATGAT	GATGAGCCAGACTTCTGTTGC
*Human P‐gp*	GCTCCTGACTATGCCAAAGC	TCTTCACCTCCAGGCTCAGT

### 4.10 Enzyme‐linked immunosorbent assay

The levels of Aβ_1‐40_, Aβ_1‐42_, IL‐1β, TNF‐α, and Klotho were measured by ELISA kit (Cusabio Biotechnology) as we described previously (Zeng et al., 2019). The level of soluble LRP1 (sLRP1) in serum was examined using an ELISA kit (USCN Life Sciences) according to the manufacturers' instructions.

### 4.11 Western blotting

The total proteins were extracted by RIPA buffer (Beyotime). Equal amounts of protein samples were separated by SDS‐PAGE and then transferred to a PVDF membrane (Millipore) as we described previously (Zeng et al., 2019). The primary antibodies we used were shown in Table 1.

### 4.12 Cell transfection

Klotho knockdown in HCPEpiCs was achieved by infection with the lentivirus (shNC, 8 × 10^7^ TU/ml, shKlotho, 6 × 10^7^ TU/ml) at a multiplicity of infection (MOI) of 80. HitransG P (GeneChem Company) was used to increase the infection efficiency. 24 h after infection, the medium was refreshed. The cells were screened with puromycin (1 μg/ml) for 1 week and harvested to examine the Klotho mRNA and protein levels.

### 4.13 Establishment of in vitro model of human BCSFB

The model was constructed with some modification following a protocol previously established (Dinner et al., 2016; Zheng & Zhao, 2002). Briefly, normal and lentivirus infected HCPEpiCs (1 × 10^5^ cells/well) were seeded on the lower side of 24‐well collagen‐coated transwell membrane filter (Corning), respectively. 48 h later, the medium was refreshed every day.

### 4.14 Transepithelial electrical resistance analysis

The barrier forming capacity of HCPEpiCs was evaluated by measuring the transepithelial electrical resistance (TEER) values using Millicell‐ERS (Millipore) for consecutive 6 days after seeded. The TEER value of the HCPEpiCs monolayer was obtained as previously reported (Dinner et al., 2016). Cell layers showing TEER values between 30 and 40 Ω · cm^2^ were used for the following experiments.

### 4.15 Paracellular permeability analysis

The permeability of HCPEpiCs monolayer was determined as described with some modification (Zhu, Su, Fu, & Xu, 2018). Briefly, the medium in the upper chamber (apical side) was replaced with 200 μl RB‐dextran (40 kDa; Ruixi Biological Technology) solution (1 mg/ml) in Hank's balanced salt solution (HBSS), and the medium in the lower chamber (basolateral side) was replaced with 1000 μl HBSS. 100 μl samples were taken from the lower chamber at 10 min intervals for 120 min. While sampling each time, the lower chamber was replenished with equal volume of HBSS. The corresponding dextran concentrations were determined by measuring fluorescence intensity using a fluorescence plate reader (Thermo Scientific) at excitation/emission wavelength of 550 nm/620 nm. The permeability coefficients of RB‐dextran across BCSFB monolayer was calculated as described (Zhu, Su, et al., 2018).

### 4.16 Analysis of Aβ transport across the in vitro human BCSFB model

The transport of soluble Aβ_1‐42_ across the BCSFB monolayer was tested in both directions [from the apical to the basolateral (A → B) and from the basolateral to the apical (B → A)] for 120 min. 0.5 μM FITC‐labeled Aβ_1‐42_ (ChinaPeptides) was used in this experiment. Fluorescence intensity was measured at excitation/ emission wavelength of 492 nm/520 nm. The apparent permeability coefficients and efflux rate of Aβ_1–42_ was quantified as described (Guo et al., 2016; Zhu, Su, et al., 2018).

### 4.17 Statistical analysis

SPSS 20.0 software was used for the statistical analyses. Except that the alternation in Y‐maze test and escape latency in Morris water maze test were analyzed using Mixed‐design ANOVA with Bonferroni‐Holm test, other in vivo data were analyzed using two‐way ANOVA with Bonferroni‐Holm test. The in vitro data were analyzed using one‐way ANOVA with LSD's test. Kruskal–Wallis test was used when variance was uneven. Values of *p* < 0.05 were considered statistically significant.

## CONFLICT OF INTEREST

The authors declare no conflict of interests.

## AUTHOR CONTRIBUTIONS

J‐R Du conceptualized this study. J‐R Du and X‐H Li designed research. Y Zhao, C‐Y Zeng, and T‐T Yang performed research. Y Zhao and C‐Y Zeng analyzed data. X Kuang assisted in some acquisition of data. C‐Y Zeng, Y Zhao, X‐H Li, and J‐R Du prepared the manuscript. J‐R Du revised the manuscript.

## Data Availability

The authors declare that the authors provide all data included in this study upon request when there is a reasonable request.
