# Supplementary figures and images for: Klotho overexpression improves amyloid‐β clearance and cognition in the APP/PS1 mouse model of Alzheimer's disease
Source: Aging Cell. 2020 Sep 21;19(10):e13239. doi: 10.1111/acel.13239 (PMC7576297; doi:10.1111/acel.13239)

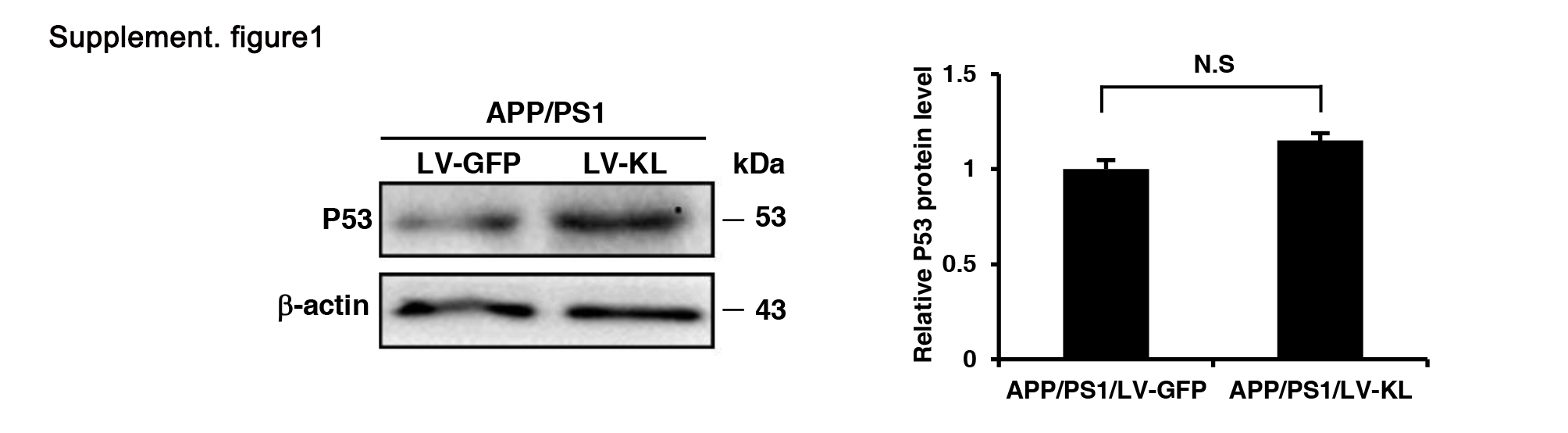

Supplement: Supplementary file 1 [file ACEL-19-e13239-s001.tif]
